# Etiology Model of Kawasaki Disease and Multisystem Inflammatory Syndromes: Mast Cell Activation

**DOI:** 10.3390/cimb48050508

**Published:** 2026-05-14

**Authors:** Darrell O. Ricke

**Affiliations:** Molecular BioInsights, 37 Pilgrim Drive, Winchester, MA 01890, USA; doricke@molecularbioinsights.com; Tel.: +1-781-866-7625

**Keywords:** Kawasaki disease, multisystem inflammatory syndrome, MIS, vasculitis disease, mast cells, histamine, vaccines, immunization, manufacturing contaminant

## Abstract

Background/Objectives: Kawasaki’s disease (KD) is a leading cause of heart disease in children. The multisystem inflammatory syndrome (MIS) associated with the SARS-CoV-2 virus is similar to KD. The etiologies of KD and MIS are unknown. Both diseases are associated with pathogens and immunizations. Methods: The Vaccine Adverse Event Reporting System (VAERS) was retrospectively examined for etiology insights into both KD and MIS. Results: Statistically significant, elevated AE MIS safety signals were observed for several COVID-19 Pfizer-BioNTech manufacturing lots. Elevated AE MIS normalized frequencies were observed in children of all ages. Immediate-onset AE KD safety signals were detected for specific vaccines and coadministered combinations of these vaccines (including specific live, attenuated virus vaccines and other specific vaccines) for young infants; a subset of these safety signals has a male sex bias, whereas others appear to be unbiased. Conclusions: Both KD and MIS are hypothesized to involve two activation pathways. The first pathway is hypothesized to involve high titers of immune complexes that activate Fc receptors on mast cells, platelets, and other immune cells. Immune complex titers higher than primary immune response levels are hypothesized to be required to activate low-affinity *IgG* *FcγR2α* receptors on immune cells and platelets. IVIG treatment is hypothesized to directly compete with immune complex binding to *FcγR2α* receptors. The second hypothesized pathway is proposed to directly activate mast cells and other immune cells without involving immune complexes and Fc receptors; lack of Fc receptor competition by immune complexes is hypothesized as a possible explanation for IVIG nonresponders for KD and MIS, worthy of future studies. The proposed etiology models for both KD and MIS may be consistent with being novel mast cell activation syndromes (MCAS). MIS is hypothesized to be KD-associated with the SARS-CoV-2 virus or the COVID-19 spike protein (MIS-V).

## 1. Introduction

Kawasaki disease (KD) (also known as mucocutaneous lymph node syndrome) is a form of vasculitis in which medium-sized blood vessels become inflamed throughout the body. KD primarily affects children under 5 years of age. Symptoms include fever, rash, conjunctivitis (red eye), oral changes (red, dry, cracked, or fissured lips, “strawberry tongue”, and inflamed oral mucosa), palmar and plantar erythema (redness of hands and feet), cervical adenopathy (enlarged lymph nodes of the neck), coronary artery aneurysms (CAAs) or lesions (CALs) (~25%), and peripheral artery aneurysms [1]. KD fever typically lasts for more than five days and is unresponsive to paracetamol (acetaminophen) or ibuprofen. KD is the leading cause of acquired heart disease (including myocarditis and CAA) in children. The skin on the hands and feet may peel after the patient’s recovery. The etiology of KD is currently unknown. Atypical (or incomplete) KD patients do not fulfill the complete diagnostic criteria for KD but are also at risk for developing coronary artery abnormalities [2]; treatment of KD patients and atypical KD patients with intravenous immunoglobulin (IVIG) and aspirin greatly reduces the incidence of CALs in patients (overview [2]). Notably, aspirin is normally contraindicated for children because of the possible risk of Reye’s syndrome [3]. Up to 20% of IVIG-treated patients develop recurrent or persistent fever (IVIG-resistant) (overview [2]). Kawasaki disease shock/toxic-shock syndrome (KDSS) is an acute phase of KD [4].

Associations between KD and multiple viruses [5,6,7,8,9,10,11,12,13,14,15,16,17,18,19,20,21,22,23,24,25,26] and bacterial pathogens [14,27,28,29,30,31,32] have been reported. KD cases frequently occur several weeks after pathogen outbreaks [22,33,34]. Pyroptosis is a form of inflammatory, programmed, and lytic cell death triggered by infections or other signals where cells rupture and release some proinflammatory molecules. Infection-triggered pyroptosis [35] and endothelial cell pyroptosis may play a role in some KD patients [36]. Seasonal exposure patterns are associated with some KD patients aged 3 years or older but not younger [37]. During the COVID-19 pandemic, the incidence of KD cases decreased and remained low during the period of masking and school closures for older children more than for infants [38,39]. KD has also been reported as a rare adverse event associated with individual vaccines and concomitantly administered vaccine combinations [40,41,42,43,44,45,46,47,48,49,50,51,52,53,54]. Patients with KD can also have altered gastrointestinal microbiota [55,56]. KD cases also temporally cluster [57]. Environmental exposures may be triggering some KD cases [58]. Cumulative prenatal and postnatal air pollution exposure to carbon monoxide (CO), nitric oxide (NO), nitric dioxide (NO_2_), and nitrogen oxide (NO_x_) but not ozone (O_3_) exposure has a dose-dependent effect on increasing KD incidence [59]. O_3_, but not CO, NO_2_, particulate matter with an aerodynamic diameter <10 μm (PM_10_), and SO_2,_ were not found to be associated with each other in a different study [60]. A study of CO, NO_2_, SO_2_, O_3_, PM_2.5_, and PM_10_ reported positive associations for only SO_2_ and PM_2.5_ for KD [61]. An exposure dosage relationship between PM_2.5_ and KD has been reported [62]. Additionally, increases in the monthly mean temperature and dry season were associated with increased KD in the Philippines [63]. PM_2.5_, PM_10_, SO_2_ (warm season), and temperature associations have been detected [64]. A meta-analysis revealed both prenatal and postnatal associations between ambient air pollution and KD [65]. KD associations include pathogens, vaccines, air pollution, and increased temperature.

Individuals with COVID-19 can develop multisystem inflammatory syndrome (MIS) in children (MIS-C), adults (MIS-A) [66], and neonates (MIS-N), with significant similarities to KD or KDSS [67]. MIS-C has also been named Pediatric Inflammatory Syndrome temporally associated with SARS-CoV-2 infection (PIMS-TS) [68]. MIS is thought to be distinct from KD because of differences in patient age profiles; gastrointestinal and cardiovascular system involvement (including myocarditis, transient left ventricular dysfunction, and depressed cardiac output); and laboratory findings [67,68,69,70,71]. Elevated troponin and elevated B-type natriuretic peptide are key laboratory findings of MIS compared with KD [72]. KD and KDSS are associated with coronary artery pathologic changes and long-term cardiovascular sequelae [73,74,75]. KD and MIS symptoms overlap with those of mast cell activation syndromes (MCAS) [76]. Note that temperature changes and air pollution are known to trigger MCAS. KD and MIS are generally considered distinct diseases.

Platelet activation plays an important role in KD pathogenesis [77]; monocyte-platelet aggregates (MPAs) (markers of platelet activation) are significantly elevated in the acute stages of KD [77]. Platelet count and plateletcrit (PCT) were found to be diagnostic markers for KD [78]. Thrombocytopenia has been reported in a KD patient [79]. Thrombocytopenia or thrombocytosis can be associated with KD [80]. In a murine model of KD vasculitis, platelets exacerbated cardiovascular inflammation [81]. A KD etiology model in which activated mast cells and platelets are important KD pathogenic characteristics has been proposed [82]. In KD, platelets and activated monocytes can result in Kawasaki disease complicated with macrophage activation syndrome (KD-MAS) [83].

In this study, the Vaccine Adverse Event Reporting System (VAERS) was retrospectively examined to obtain additional insights into the pathogenesis of both KD and MIS. Previously proposed KD and MIS etiology models are refined, linking pathogen infections, immunization, and environmental triggers with activated mast cells. Hypothesized unknown microbial manufacturing contaminants and live, attenuated vaccine viruses are candidates for future studies of KD and MIS post-immunization.

## 2. Materials and Methods

This is a retrospective analysis of the VAERS database [84] from 1 January 1990, until 30 January 2026. The VAERS database was searched for “Kawasaki’s disease”, “Multisystem inflammatory syndrome”, “Multisystem inflammatory syndrome in children”, “Multisystem inflammatory syndrome in adults”, and Death AEs. The Ruby program vaers_slice5.rb [85] was used for retrospective analysis of the VAERS data files VAERSDATA, VAERSSYMPTOMS, and VAERSVAX for the years 1990–2026 and NonDomestic.

For each vaccine (*V_name_*) and each adverse event (*X*) in VAERS for the selected age group, normalized AE frequencies per *P* = 100,000 VAERS reports per category of AEs can be calculated with Equation (1) (the count *X* for *V* for ages selected over the population count of all individuals immunized for *V* for the ages selected).

(1) $$AE\left(V|X,P\right)\;normalized\;frequency=\frac{AE(V|X,P)}{P_{age}^{V}}\times P_{100,000}$$

Vaccines and vaccine combination names including the text “no brand name”, “foreign”, “unknown”, and “vaccine not specified” were excluded to avoid possible reporting biases due to the possibility of underrepresentation of less severe AEs resulting in increased normalized frequency estimates. Data was selected for a minimum of five AE reports per vaccine or coadministered vaccines. Data for vaccines with at least 100 VAERS reports for the selected population age group were selected ($P_{age}^{V}\;\geq 100$). For reference, the names of coadministered vaccines are joined with the plus symbol. Microsoft Excel was used to create figures. An online calculator for the Chi-square test 2 × 2 contingency table was used [86].

## 3. Results

**Figure 1:**
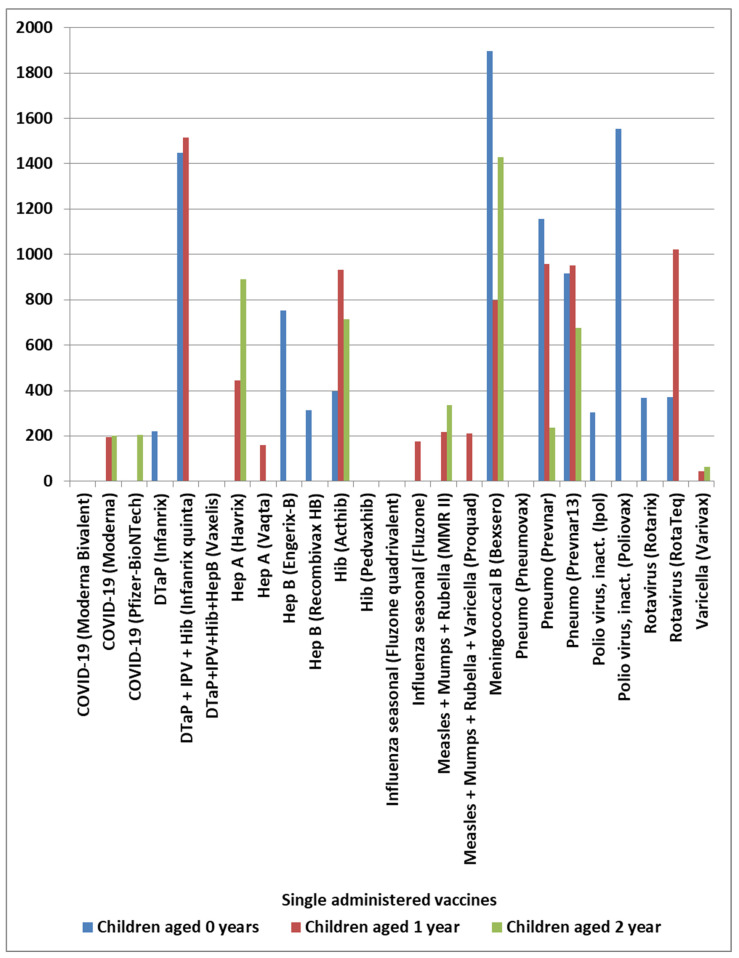
Kawasaki disease normalized frequency for singly administered vaccine by child year of age 0, 1, and 2.

**Figure 2:**
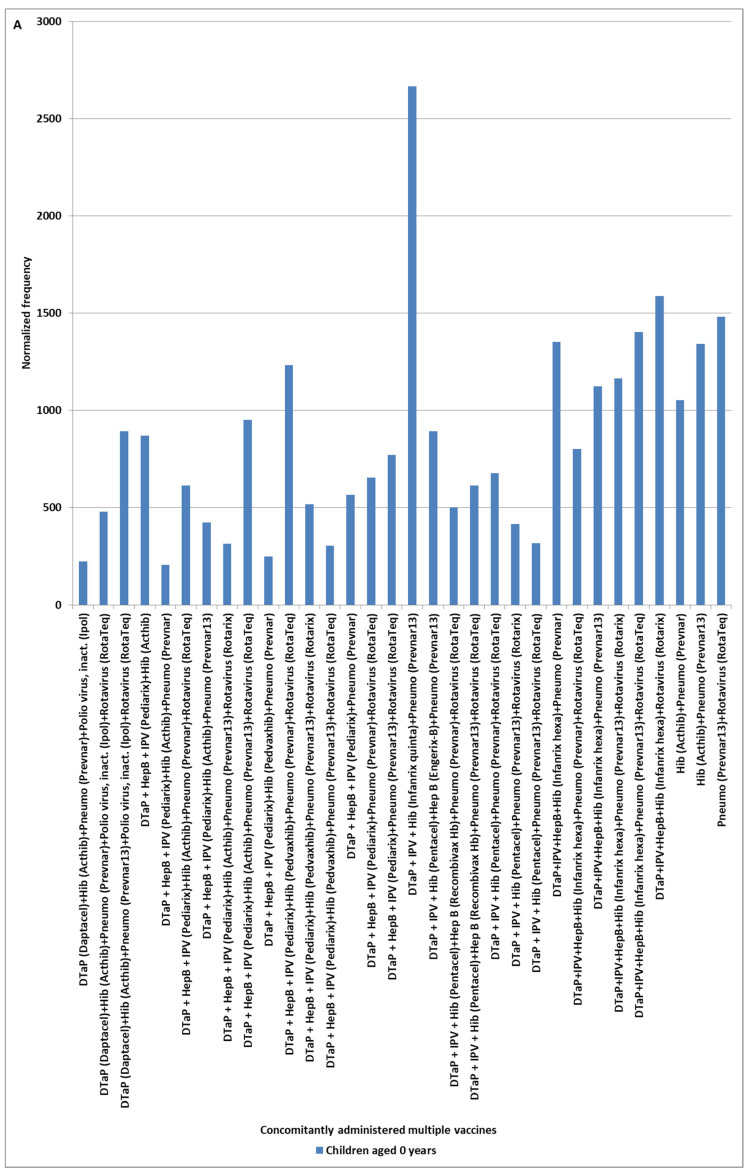

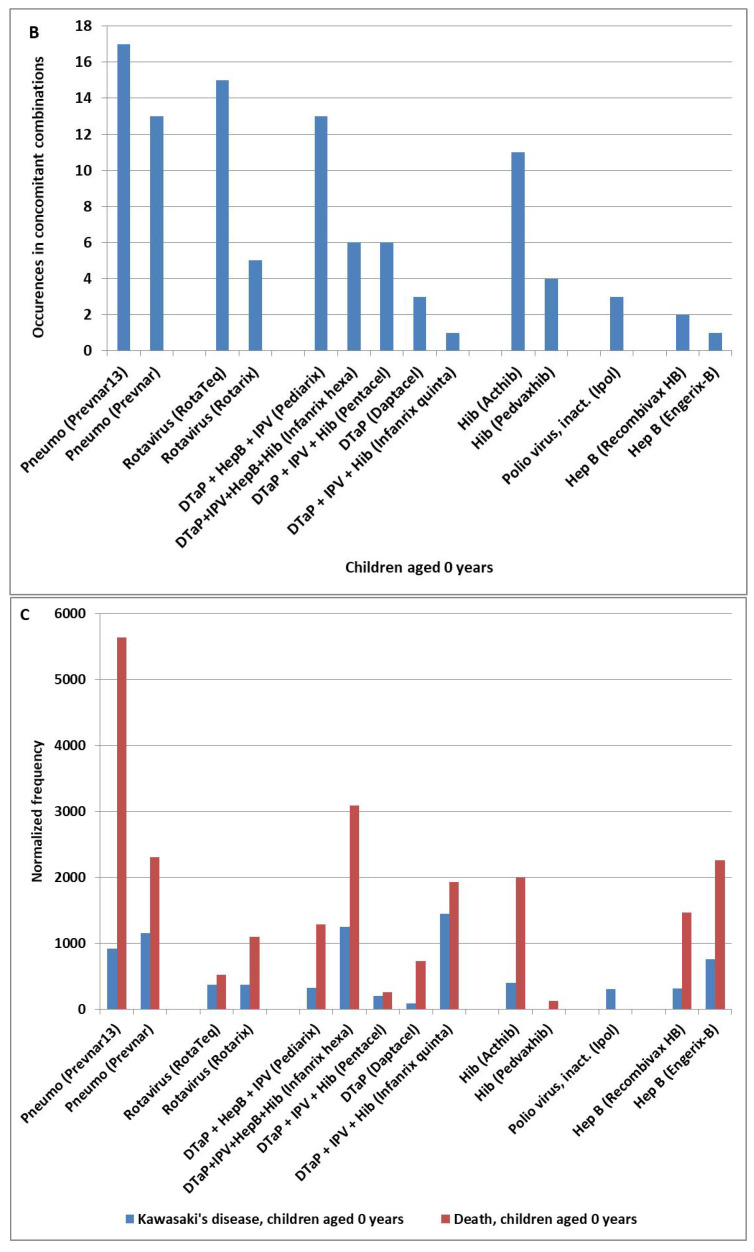
Kawasaki disease normalized frequency for infants aged 0 (**A**) concomitantly administered vaccines, (**B**) number of occurrences in concomitantly administered vaccines, and (**C**) with AE death (Pearson r = 0.66).

In VAERS, AE KD was observed with elevated safety signals for multiple specific vaccines with different normalization frequencies for children aged 0, 1, and 2 years (Figure 1); a subset of these vaccines was observed with elevated normalized frequencies for infants aged 0 years (Figure 2A). The frequency of vaccine names in Figure 2A is plotted in Figure 2B; these same vaccines have similar patterns of normalized frequencies for AE death, Pearson r = 0.66 (Figure 2C). Two commonalities were observed for these vaccines. First, six of the 30 identified vaccines are live, attenuated virus vaccines: Measles + Mumps + Rubella (MMR II) and (Priorix), Measles + Mumps + Rubella + Varicella (Proquad), rotavirus (RotaTeq) and (Rotarix), and varicella (Varivax). Second, 18 of the other 24 identified vaccines include bacteria and/or *Saccharomyces cerevisiae* (baker’s yeast) in the vaccine manufacturing process. As a possible cross-check of identified safety signals, the yearly normalized frequencies are illustrated in Table 1 for vaccines with at least 75 KD AEs.

Coadministration of two or more vaccines with identified normalized frequency safety signals may result in higher normalized frequencies than for individual vaccines. Across all ages, two concomitantly administered vaccine combinations were observed with more AE KD: DTaP + IPV + HepB + Hib (Infanrix hexa) + Pneumo (Prevnar13) (brand name Prevnar 13) at 1077 and DTaP + IPV + HepB + Hib (Infanrix hexa) + Pneumo (Prevnar13) + Rotavirus (Rotarix) at 1760; these normalized frequencies can be compared to the sum of the normalized frequencies for individual vaccines: DTaP + IPV + HepB + Hib (Infanrix hexa) at 1235, Pneumo (Prevnar13) at 220, and Rotavirus (Rotarix) at 538 (observed 1077 vs. sum = 1454 and observed 1760 vs. sum = 1992). With less supporting data, related combinations also exhibit higher normalized frequencies for vaccine combinations: DTaP + IPV + Hib (Infanrix quinta) + Pneumo (Prevnar13) + Rotavirus (Rotarix) at 1786 andDTaP + IPV + Hib (Infanrix quinta) + Pneumo (Prevnar13) + Rotavirus (Rotarix) at 5634 with DTaP + IPV + Hib (Infanrix quinta) at 1418 (observed 1786 vs. sum = 1638 and observed 5634 vs. sum = 2176).

**Figure 3:**
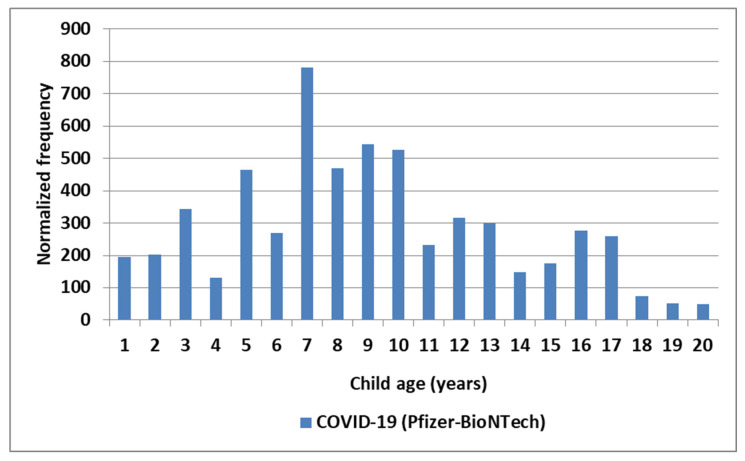
MIS normalized frequency by age for COVID-19 (Pfizer-BioNTech).

**Figure 4:**
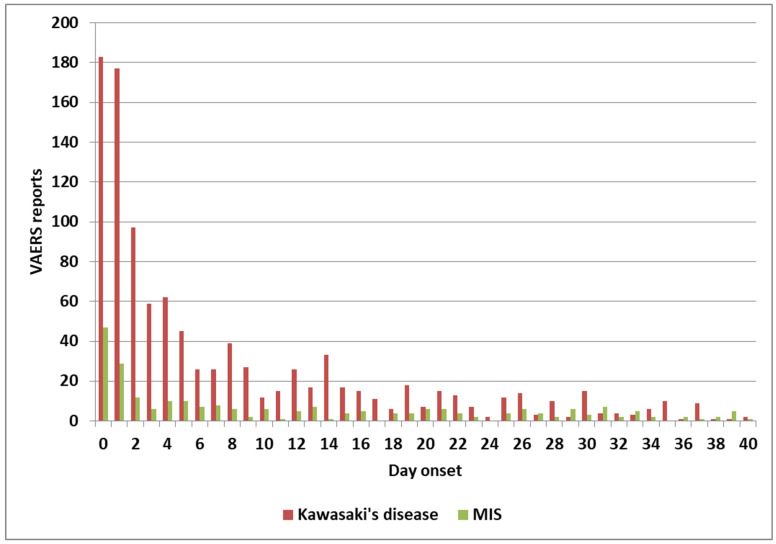
Kawasaki disease and MIS onset day (Pearson r = 0.90).

**Figure 5:**
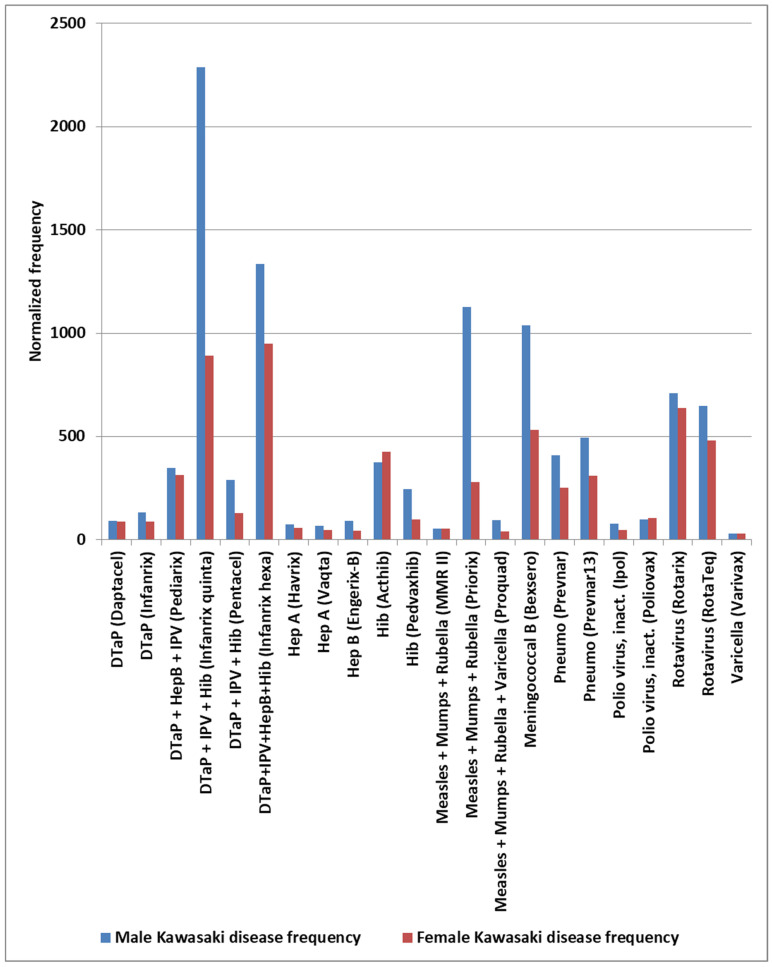
Kawasaki disease normalized frequency by sex.

**Figure 6:**
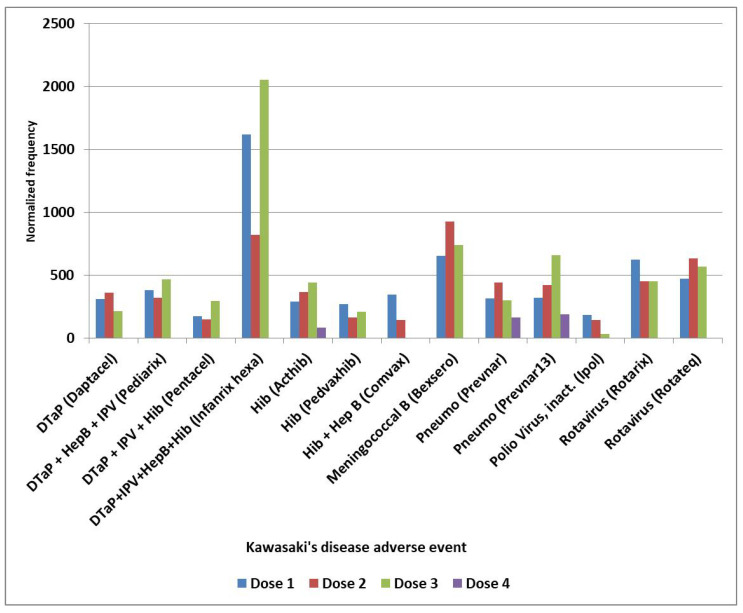
Kawasaki disease adverse events normalized frequency by vaccine dose.

The normalized frequencies for MIS-V observed for the COVID-19 Pfizer-BioNTech vaccine are shown in Figure 3. While events reported to VAERS are subject to reporting bias with fewer reports with increased time since immunization, the highest reports for KD and MIS are 1–2 days, with possible small increases associated with antibody immune responses (Figure 4). Note that the day of onset patterns post-immunization for KD and MIS correlate with Pearson r = 0.90 (Figure 4). An increased male sex bias is known for KD; normalized frequencies for AE KD by sex in VAERS are illustrated in Figure 5. Four vaccines have higher imbalances between normalized frequencies for males versus females: DTaP + IPV + Hib (Infanrix quinta), DTaP + IPV + HepB + Hib (Infanrix hexa), Measles + Mumps + Rubella (Priorix), and Meningococcal B (Bexsero) (Figure 5), whereas some vaccines have roughly equivalent normalized frequencies (Figure 5). Note that Measles + Mumps + Rubella (MMR II) has low normalized frequencies similar to Measles + Mumps + Rubella + Varicella (Proquad) but discordant from Measles + Mumps + Rubella (Priorix) with higher normalized frequencies for females and much higher normalized frequencies for males (Figure 5). Specific immunization doses were observed with elevated AE KD normalized frequencies (Figure 6). For MIS, more of the reports were observed for the second COVID-19 shot. For COVID-19 Pfizer-BioNTech, the normalized frequency is higher for the second shot (169 reports) versus the first shot (155 reports). The symptoms reported in the VAERS for KD and MIS patients are summarized in Table S1.

**Figure 7:**
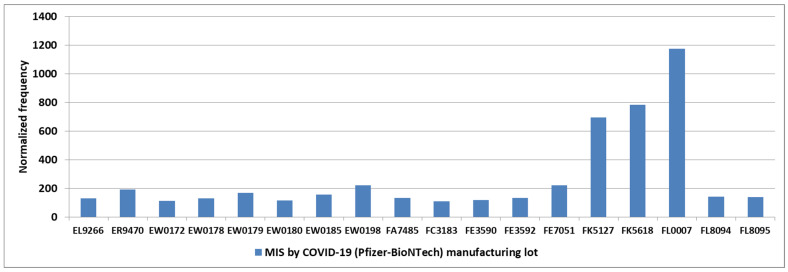
MIS normalized frequency by COVID-19 (Pfizer-BioNTech) manufacturing lot (minimum 1000 AEs by lot and AE MIS ≥ 2).

For Kawasaki’s disease, five vaccines have manufacturing lots with three reported Kawasaki’s disease cases: DTaP + HepB + IPV (Pediarix): AC21B248CA, Hib (Acthib): T1E12, Pneumo (Prevnar13): EG8873 and CS7258, Rotavirus (Rotarix): RT014 and RT018, and Rotavirus (RotaTeq): 0324X. For MIS, seven COVID-19 (Pfizer-BioNTech) manufacturing lots have four or more MIS cases: EW0179: 4 reports, FE7051: 4 reports, FG6273: 4 reports, FK5127: 24 reports, FK618: 20 reports, FL0007: 21 reports, and FN4072: 6 reports (Figure 7). Thirty-four COVID-19 Pfizer-BioNTech lots with at least 1000 AEs had 1 AE MIS with an average normalized frequency of 57.7, SD = 20.6. For the following chi-square comparisons, these 34 lots have an average of 2082 AEs per lot. The four vaccine lots with the most MIS reports include lot FK5127 with a normalized frequency of 696 (24 of 3448 VAERS reports, χ^2^ = 0.000525), FK5618 at 782 (20 of 2255, χ^2^ = 0.000223), FL00007 at 1173 (21 of 1790, χ^2^ = 0.000004), and FN4072 at 1775 (6 of 338, χ^2^ = 0.000000) (Figure 7). The elevated MIS normalized frequencies for manufacturing lots are not known to be associated with high-risk recipient groups or background occurrences.

## 4. Discussion

Candidate KD safety signals were identified for specific vaccines (Figure 1). Considering KD AEs by year (Table 1), sex (Figure 5), and vaccine dose (Figure 6), it provides possible insights into the reproducibility of identified KD safety signals (Figure 1 and Figure 2). The yearly normalized frequencies are illustrated in Table 1 for vaccines with at least 75 KD AEs. No KD AEs were reported for the years 1995 to 2002 for the Hib (ActHib) vaccine. Observed yearly variability may be associated with data sampling size, possible changes to manufacturing processes, or other causes.

The initial etiology model for KD and MIS is for high titers of IgG antibodies in immune complexes binding to low-affinity FcγR2α receptors, activating mast cells, platelets, and other immune cells [82]. VAERS results for this study support expanding this etiology model to also include activation of mast cells from live, attenuated vaccine viruses or unknown vaccine component including possible contaminant(s) for specific vaccines (Figure 1, Figure 2 and Figure 7). Endotoxin, a possible COVID-19 vaccines manufacturing contaminant from *Escherichia coli*, exposure is known to activate mast cells [87]. The limulus amebocyte lysate (LAL)-based assays may miss endotoxins (e.g., low endotoxin recovery (LER)) due to a “masking effect” caused by chelators or detergents commonly used in buffer formulations [88]. Microbial components are known to activate mast cells and immune responses via multiple mechanisms. Note that the SARS-CoV-2 spike protein binds to bacterial LPS (endotoxin) and boosts proinflammatory activity [89,90]. By design, vaccines stimulate innate and humoral immune responses. The *Toll-like receptor* (*TLR4*) is activated by LPS (endotoxin) of Gram-negative bacteria [91]. TLRs recognize pathogen-associated molecular patterns (PAMPS). KD is associated with pathogen-associated molecular patterns (PAMPS) [92] and microbe-associated molecular patterns (MAMPS) [32]. Increased TLR2 and TLR4 expression in peripheral neutrophils has been detected in some KD patients [93]. Some KD cases may be associated with endotoxins and elevated soluble CD14 (sCD14) [94,95]. Polyclonal expansion of TCRBV2- and TCRBV6-bearing T-cells occurs in KD patients (likely associated with endotoxin exposure) [31]. Low-level endotoxin induces potent inflammatory activation of human blood vessels [96].

Multiple patients with KD or MIS also have associated gastrointestinal (GI) symptoms/intestinal involvement [97,98]; this may include intestinal dysbiosis and sometimes disruption of the gut barrier [99]. Disruption of the gut barrier is likely associated with the presence of a superantigen [100]**.** Elevated Vβ2 T-cells expansion in some KD patients is consistent with the superantigen model [101]. KD patients with abdominal manifestations (symptoms) are more likely to be IVIG-resistant (*p* < 0.005) and have CAA (*p* = 0.007) [102].

### 4.1. KD and MIS Etiology Model

Etiology Model: KD and MIS are associated with activated mast cells and platelets [82]. Pathogen-associated KD and MIS cases are hypothesized to be triggered by immune complexes binding to low-affinity IgG receptors on mast cells, platelets, and other immune cells [82]. KD and MIS disease delayed cases following pathogen surges by weeks (Figure S1) have been hypothesized to be due to an envisioned threshold for sufficient immune complex binding to low-affinity IgG receptors [82]. Standard IVIG treatment has been hypothesized to compete with immune complexes binding of these low-affinity IgG receptors [82]. CAAs and cardiac symptoms for some KD and MIS patients are hypothesized to be associated with cardiac capillary vasoconstrictions [103,104].

**Hypothesis** **H1.** *Disruption of the gut barrier in some KD and MIS patients and resulting exposures to microbial components may be triggering disease, including activation of mast cells; this can occur for either persistent GI infections or immunizations with live, attenuated virus vaccines like rotavirus vaccines. Unlike pathogen-associated delayed disease onset, disease onset may be rapid (within days of immunization).*

**Hypothesis** **H2.** *Similar to observed COVID-19 Pfizer-BioNTech manufacturing lots with elevated MIS safety signals, other identified vaccines (not live, attenuated virus vaccines) with immediate onset KD safety signals may be associated with possible unknown microbial manufacturing contaminants.*

**Hypothesis** **H3.** *The KD bias towards children ages 0–5 is hypothesized to be partially attributed to observed KD-V AEs.*

**Hypothesis** **H4.** *Multisystem inflammatory syndrome is Kawasaki disease associated with the SARS-CoV-2 pathogen, with differences associated with specific infectious pathogen (e.g., SARS-CoV-2). Similarly, MIS-V is KD-V [105] associated with a COVID-19 (spike protein) vaccine. The differences between KD and MIS are proposed to be associated with the SARS-CoV-2 virus symptoms (MIS-C, MIS-A, and MIS-N).*

Pathogen-associated reports are hypothesized to be associated with elevated immune complexes IgG antibody levels above primary immune response levels, activating low-affinity IgG *FcγR2α* receptors on platelets, mast cells, and additional immune cells (note the risk of persistent infections) (Table 2) [104,106]. Elevated histamine and likely serotonin levels are likely associated with most of the KD and MIS symptoms [82]. For KD and MIS patients with high IgG antibody titers, IVIG treatment is hypothesized to directly compete with immune complex binding to *FcγR2α* receptors, resulting in reduced activation of mast cells and platelets, and relief of associated symptoms. Immunization and environmental exposures can activate mast cells, immune cells, and likely platelets without (likely IVIG-resistant) or sometimes with *FcγR2α* receptor binding (e.g., humoral responses post-immunization) (Table 2). Gastrointestinal symptoms are reported in the majority of MIS-C patients [107,108,109,110,111]. A MIS-A patient with profound gastrointestinal symptoms has been reported [112]. SARS-CoV-2 virus or spike protein (COVID-19 vaccines) can induce additional gastrointestinal and cardiac symptoms in MIS and MIS-V patients, respectively. The spike protein also activates mast cells via *TLR4* and *angiotensin-converting enzyme 2 *(*ACE2*) receptors [113]. For KD and MIS associated with onset within a few days of immunization (Figure 4), hypothesized unknown microbial manufacturing contaminant(s) (or spike protein binding) is hypothesized to activate mast cells via TLR4; this activation pathway does not involve *FcγR2α* receptors, and these patients are anticipated to be resistant to IVIG treatment (Table 2).

Notably, overall immune activation is increased in KD [114]. It is unknown whether histamine intolerance (HIT) plays a role in KD or MIS. Multiple factors can influence an individual’s tolerance threshold for histamine, including drugs [115], foods (cocoa, spinach, tomatoes, wine, beer, cheeses, yogurt, meat, soy, fermented foods, etc.) [115,116], the gastrointestinal microbiome [115], and the stage of the menstrual cycle [116].

### 4.2. Age-Related Risk Patterns

The proposed KD and MIS etiology model proposes the activation of mast cells, platelets, and immune cells by Fc receptor binding to immune complexes or via direct activation of immune cells. Maternally transferred antibodies (matAbs) may play a role in KD-N and MIS-N in neonates with neonate antibody responses combined with matAbs to reach the envisioned higher levels of IgG antibodies in immune complexes needed to trigger disease [117]. For the 0–5 year age group, it appears that specific vaccines either contain live, attenuated vaccine viruses or (based on elevated MIS for multiple COVID-19 Pfizer-BioNTech manufacturing lots) hypothesized unknown microbial manufacturing contaminant(s); these observations are worthy of follow-up studies for AE KD (KD-V) (Figure 1) and also AE MIS (MIS-V) (Figure 3). Associations of KD with immunization (Figure 1) may account for the lack of seasonal exposure patterns for some KD patients aged younger than 3 years [37]. The normalized frequencies observed for COVID-19 (Pfizer-BioNTech) may approximate MIS (both MIS-C and MIS-V) risk levels in children (Figure 3).

### 4.3. Cardiac Adverse Events and Acquired Heart Disease

This etiology model also hypothesizes that aneurysms are pressure-induced by contracted cardiac capillary pericyte vasoconstrictions [104]; notably, serotonin released from activated platelets is also associated with vasoconstrictions [118,119]. Induced cardiac capillary pericyte contractions are hypothesized to be associated with anoxia and possibly pressure-induced CAA and peripheral artery aneurysms [103,104]. Untreated patients with ongoing ischemia are hypothesized to experience cardiac myocyte anoxia, which may account for KD-associated acquired heart disease; this also explains the vascular dysfunction in patients who do not have echocardiographic evidence of coronary artery abnormalities in the acute phase of KD. An increased proportion of KD patients with CAA also have the plasma fibrinogen (FG) alpha genotype Thr312Ala [120]. Sex differences in cardiac mast cells activation have also been observed [121]; this may be associated with the KD male sex bias for specific vaccines (Figure 5).

The differences between MIS-related cardiac symptoms and KD-related symptoms (myocarditis, transient left ventricular dysfunction, and depressed cardiac output) may be directly due to the SARS-CoV-2 virus or the SARS-CoV-2 vaccine spike protein. For COVID-19 mRNA vaccines, circulating spike proteins are observed in vaccinees with myocarditis [122], along with elevated cardiac troponin levels [123]. For COVID-19 vaccines, the spike protein disrupts cardiac pericytes through *cluster of differentiation 147* (*CD147*) receptor-mediated signaling and another unknown mechanism [124]. The spike protein also activates mast cells via *TLR4* and *angiotensin-converting enzyme 2 *(*ACE2*) receptors [113]. These spike protein interactions may account for the increased risk for myocarditis and transient left ventricular dysfunction observed in MIS compared with KD [125]. Note that the spike protein interactions cannot account for the MIS normalized frequency disparities for COVID-19 Pfizer-BioNTech manufacturing lots (Figure 7).

### 4.4. KD and MIS Delayed Onset

Clusters of KD and MIS (Figure S1) reports are frequently observed with delayed disease onset (approximately 1 month or more) following various pathogen [22,33,34] and COVID-19 outbreaks [126], respectively. For these delayed disease onset patterns, the proposed etiology model requires IgG antibody levels to be higher than primary immune response levels to trigger disease [117]. One scenario includes persistent infections (e.g., gastrointestinal infections), which may occur in some KD and MIS patients [117]. Elevated SARS-CoV-2 antibody titers [107,127,128,129], current SARS-CoV-2 infections, or prior SARS-CoV-2 infections or exposures [130,131,132,133,134,135] are observed in MIS patients. For MIS-C, sustained levels of inflammatory macrophage-activating, Fc receptor-binding antibodies are selectively maintained in severe disease [136]. MIS-C develops in some children with COVID-19 and persistent SARS-CoV-2 infections [137].

### 4.5. KD Genetics

Genetic variants are predicted to increase or decrease associated with KD and MIS risks. Confirmed KD genetic variants include *inositol 1,4,5-trisphosphate 3-kinase C (ITPKC)* [138,139], *caspase-3 (CASP3)* [139,140], *toll-like receptor 6* (***TLR6***) [141], and the low-affinity IgG receptor gene *FcγR2α* (encoding *FcγRIIa*) [142,143,144,145] (Table 3). The *FcγR2α* rs1801274 C allele encodes arginine (R) (low binding to IgG2 and IgG3), and the T allele encodes histidine (H) (high binding to IgG2 and IgG3) [146]. The *FcγR2α* pHis167Arg is associated with KD risk in males [147]. Candidate KD-associated genes are associated with the immune system, calcium signaling, KD susceptibility, IVIG resistance, and aneurysm formation (reviewed [148,149]) (Table 3). Note that *CASP3* is released by activated mast cells [150]. Mast cells express *CD40* ligand (*CD40L*) that interacts with *CD40* on B-cells [151]. While no association with the *FcγR2α* rs1801274 polymorphism was found, MIS-C patients with the homozygous *FcγR2α* rs1801274 gene polymorphism developed severe cardiac dysfunction [152]. Individual genetics alter KD and MIS risks. Genetic variants in T helper cell pathways may contribute to immune dysregulation in KD [153]. Identified genetic variants associated with KD play roles in immune cells, including mast cells, activation or signaling.

### 4.6. IVIG Treatment and IVIG Resistance

The model of high levels of IgG antibody binding low-affinity IgG *FcγR2α* receptor represents a potential novel form of antibody-dependent enhancement (ADE) for both KD and MIS [106]. IVIG treatment is hypothesized to compete with pathogen IgG antibodies for *FcγR2α* receptor binding, with a possible increased risk for IVIG resistance; note that TLR4 (non-*FcγR2α* receptor) activated mast cells are hypothesized to be more likely IVIG-resistant (due to a different activation pathway). This model potentially explains the unpredictable ineffectiveness of current therapy and the observed IVIG resistance in both KD and MIS patients.

### 4.7. MIS Differences from KD

KD and MIS reports not temporally associated with recent vaccinations are hypothesized to be associated with persistent (perhaps gastrointestinal in some reports) infections. The greater number of KDs at ages 0–5 is hypothesized to be partially attributed to observed KD-V AEs. Resilience against the development of pressure-induced CAA may reduce incidence rates as the age of patient increases. SARS-CoV-2 infection or spike protein interactions may account for cardiac differences between MIS and KD. Otherwise, MIS appears to be KD-associated with either the SARS-CoV-2 virus or COVID-19 immunization (MIS-V).

### 4.8. Candidate Adjunctive Treatments

If the proposed KD and MIS etiology model is correct, then additional adjunctive treatments, including mast cell stabilizers, antihistamines, and possibly serotonin antagonists are candidates for future institutional review board (IRB)-approved targeted clinical studies (e.g., report series) (perhaps targeting IVIG nonresponders).

### 4.9. Study Limitations

The VAERS database includes only a small subset of adverse events experienced by vaccinees. Any reporting biases or exclusion of adverse events would perturb the accuracy of VAERS, which represents the population.

### 4.10. Study Recommendations

This study hypothesizes that mast cell and platelet activation drive the etiology of both KD and MIS. Many of the disease symptoms are consistent with hypothesized elevated levels of histamine and/or serotonin. Evaluations of adjunctive treatments targeting elevated histamine or serotonin levels are candidates for evaluation in approved clinical studies. Early treatments may reduce the risk of CALs and acquired heart disease in KD patients and ventricular dysfunction and cardiac adverse events in MIS patients. For future studies, KD and MIS reports not associated with immunizations are hypothesized to have undiagnosed persistent infections. If future studies confirm hypothesized microbial manufacturing contaminations, elimination or reduction in these contaminants are hypothesized to reduce AEs KD safety signals in children. Modifications of current childhood live attenuated virus vaccines to be non-replicating in human cells are hypothesized to reduce AEs KD safety signals in children.

## 5. Conclusions

The etiology of both KD and MIS are both likely novel MCAS. An etiology model is proposed that can account for the etiology of both KD and MIS. For pathogen-associated infections, high-titer immune complexes are hypothesized to activate low IgG-affinity *FcγR2α* receptors; this may account for observed delayed disease onset clusters following pathogen outbreaks. Air pollution and increased temperature can also activate mast cells, triggering KD. Based on elevated safety signals observed for several COVID-19 Pfizer-BioNTech manufacturing lots, evaluation of possible unknown microbial manufacturing contaminants of specific vaccines with immediate onset KD and MIS post-immunization is worthy of future studies. Activating mast cells either directly via TLR4, PAMPS activation, MAMPS activation, or by live attenuated vaccine viruses is hypothesized for specific vaccines with immediate onset post-immunization; these patients are proposed for future studies of IVIG nonresponders. KD-related male sex bias may be partially due to observed male sex bias for multiple specific vaccines; sex differences between cardiac mast cells are worth of future studies. While appearing clinically distinct, MIS is hypothesized to be KD-associated with the SARS-CoV-2 virus or the COVID-19 spike protein (MIS-V).

## Figures and Tables

**Table 1:** Kawasaki disease normalized frequency by year for vaccines with greater than 75 VAERS KD reports. Yearly date range end years selected for a minimum of 500 total yearly AEs.

Year	Hib (ActHib)	Meningococcal B (Bexsero)	Pneumo (Prevnar)	Pneumo (Prevnar13)	Rotavirus (Rotarix)	Rotavirus (Rotateq)
1995	0					
1996	0					
1997	0					
1998	0					
1999	0					
2000	0		0			
2001	0		36			
2002	0		117			
2003	236		240			
2004	0		56			
2005	217		174			
2006	126		124			
2007	768		703			1443
2008	127		411			537
2009	103		636			577
2010	339		473	488		463
2011	872		795	310		268
2012	1370		1682	519	1356	763
2013	91		364	431	459	369
2014	875			482	482	192
2015	144			198	409	242
2016	1216	778		419	932	152
2017	610	1039		194	384	157
2018	631	1183		397	1117	398
2019	487	1256		304	885	144
2020	1409	1051		577	1581	247
2021	467	560		323	672	428
2022	1359	463		751	1362	281
2023	225	869		701	430	731
2024	374	646		897	654	500
2025	310	905		1592	712	112
Yearly average	399	875	415	537	817	421
Standard deviation	454	264	447	342	400	314

**Table 2:** Kawasaki and MIS etiology model disease factors, mast cell activators, and hypothesized IVIG resistance.

Primary Factor	Additional Factor	Mast Cell Activator(s)	Likely IVIG Resistance
neonate pathogen infection	maternally transferred antibodies (MatAbs)	high Ab titers	very low
pathogen infection	elevated Ab titers (ongoing, prior infections, …)	high Ab titers	very low
immunization	elevated Ab titers	high Ab titers	very low
immunization	hypothesized manufacturing contaminant(s)	unknown microbial components, or spike protein [113]	high
immunization	live attenuated virus vaccine GI pathogen (e.g., rotavirus)—possible disruption of gut barrier	possible high Ab titers and/or unknown microbial components	variable depending upon mast cell activators
GI infection	disruption of gut barrier [99]	unknown microbial components	high
environmental exposures including increased temperature	genetic risk factor	direct mast cell activation	high

**Table 3:** Kawasaki disease-associated genes and candidate-associated genes.

Gene	Gene Name	Pathway	References
*BLK*	*B-cell lymphoid tyrosine kinase*	regulates B-cell receptor signaling and development	[144,154,155,156]
*CASP3*	*caspase-3*	modules immune responses	[139,140]
*CD40*	*tumor necrosis factor receptor superfamily member 5*	mediates immune responses	[154,155]
*CD40L*	*CD40 ligand*, *CD154*	mediates immune responses; mast cells express *CD40* ligand (*CD40L*) that interacts with *CD40* on B-cells	[151]
*FcgR2a*	*Fc fragment of IgG receptor IIa*, *CD32*	encodes a low-affinity cell surface receptor that binds the Fc region of IgG antibodies	[142,143,144,145,157]
*FcgR2b*	*Fc fragment of IgG receptor IIb*	encodes a low-affinity inhibitory receptor for the Fc region of immunoglobulin gamma	[158]
*FcgR2c* gene copy number	*Fc fragment of IgG receptor IIc*	encodes a low-affinity inhibitory receptor for the Fc region of immunoglobulin gamma	[159]
*FcgR2c-ORF*	*Fc fragment of IgG receptor IIc open reading frame*	encodes a low-affinity inhibitory receptor for the Fc region of immunoglobulin gamma	[160]
*FcgR3b* gene copy number	*Fc fragment of IgG receptor IIIb*	encodes a low-affinity inhibitory receptor for the Fc region of immunoglobulin gamma	[159]
*HLA*	*human leukocyte antigen*	T-cell immune responses	[155]
*IGHV*	*Immunoglobulin heavy chain variable region*	B-cell antibody responses	[161]
*ITPKC*	*inositol 1,4,5-trisphosphate 3-kinase C*	calcineurin, a nuclear factor of the activated T-cell pathway—calcium signaling pathway	[138,139]
*KCNN2*	*potassium calcium-activated channel subfamily N member 2*	associated with CAA	[162]
*MYH14*	myosin *heavy chain 14*	whole-exome sequencing gene candidate	[163]
*NA1 *of *FcgR3B*	*neutrophil antigen 1*	variant overexpression in IVIG nonresponders	[157]
*ORAI1*	*calcium release-activated calcium modulator 1*	involved in calcium influx in immune cells	[164]
*RBP3*	interphotoreceptor retinoid-binding protein	whole-exome sequencing gene candidate	[163]
*SMAD3*	*mothers against decapentaplegic homolog 5*	*TGF-beta* signaling pathway	[165]
*SMAD5*	*SMAD family member 5, MADH5, JV5-1*, *mothers against decapentaplegic homolog 5*	*TGF-beta* signaling pathway	[166]
*TGFBR2*	*transforming growth factor beta receptor 2*	regulates immune cell differentiation and activation	[165,167]
*TLR6*	*toll-like receptor 6*	a pattern recognition receptor (PRR) that detects pathogens	[141]

## Data Availability

The original data presented in the study are openly available in https://doi.org/10.7910/DVN/QRBEQT, Harvard Dataverse, V2. An early version of this study was released as a preprint https://www.preprints.org/manuscript/202603.0353 (accessed on 19 Februrary 2026) and mirrored at https://sciety.org/articles/activity/10.20944/preprints202603.0353.v1 (accessed on 5 March 2026).

## Supplementary Materials

The following supporting information can be downloaded at https://www.mdpi.com/article/10.3390/cimb48050508/s1.

## Abbreviations

The following abbreviations are used in this manuscript:

ACE2	Angiotensin-converting enzyme 2
ADE	Antibody-dependent enhancement
AE	Adverse event
B-cell	Immune B lymphocyte
BLK	B-cell lymphoid tyrosine kinase
CAA	Coronary artery aneurysm
CALs	Coronary artery lesion
CASP3	Caspase 3
CD14	Cluster of differentiation 14
CD147	Cluster of differentiation 147, also known as EMMPRIN (Extracellular Matrix Metalloproteinase Inducer), or Basigin
CD40	Cluster of differentiation 40
CD40L	Cluster of differentiation 40 ligand
CO	Carbon monoxide
COVID-19	Coronavirus disease 2019
DTaP	Diphtheria, tetanus, and pertussis (whooping cough) vaccine
Fc	fragment crystallizable region of antibody
Fc*γ*R	Fc gamma receptor
FG	plasma fibrinogen
GI	gastrointestinal
Hep B	hepatitis B
Hib	*Haemophilus influenzae* type b vaccine
HIT	histamine intolerance
HLA	human leukocyte antigen
IgG	immunoglobulin G
IGHV	immunoglobulin heavy variable gene
IPV	inactivated poliovirus vaccine
ITPKC	inositol 1,4,5-trisphosphate 3-kinase C
IVIG	intravenous immunoglobulin
KCNN2	Potassium Calcium-Activated Channel Subfamily N Member 2
KD	Kawasaki’s disease
KD-MAS	Kawasaki disease complicated with macrophage activation syndrome
KD-N	Kawasaki’s disease in neonates
KDSS	Kawasaki disease shock/toxic-shock syndrome
KD-V	Kawasaki’s disease associated with vaccination
LAL	Limulus Amebocyte Lysate
LER	Low endotoxin recovery
LPS	Lipopolysaccharide
MAMPS	Microbe-associated molecular patterns
matAbs	Maternally transferred antibodies
MCAS	Mast cell activation syndromes
MIS	Multisystem inflammatory syndrome
MIS-A	Multisystem inflammatory syndrome in adults
MIS-C	Multisystem inflammatory syndrome in children
MIS-N	Multisystem inflammatory syndrome in neonates
MIS-V	Multisystem inflammatory syndrome after COVID-19 vaccination
MMR	Measles, mumps, and rubella vaccine
MPAs	Monocyte-platelet aggregates
mRNA	Messenger ribonucleic acid
MYH14	Myosin heavy chain 14
NA1	Neutrophil antigen 1
NO	Nitric oxide
NO_2_	Nitric dioxide
NO_x_	Nitrogen oxide
O_3_	Ozone
ORAI1	calcium release-activated calcium modulator 1
ORF	Open reading frame
PAMPS	pathogen-associated molecular patterns
PCT	Plateletcrit
PIMS-TS	Pediatric Inflammatory Syndrome temporally associated with SARS-CoV-2 infection
PM_10_	Inhalable particulate matter 10 μm or smaller
PM_25_	fine inhalable particles less than or equal to 2.5 μm in diameter
RBP3	retinol-binding protein 3
SAE	Serious adverse event
SARS-CoV-2	Severe acute respiratory syndrome coronavirus 2
sCD14	Soluble CD14 protein
SMAD3	Mothers against decapentaplegic homolog 3
SMAD5	Mothers against decapentaplegic homolog 5
SO_2_	Sulfur dioxide
T-cell	T lymphocyte
TCRBV2	T-cell receptor Beta-chain V2
TCRBV6	T-cell receptor Beta-chain V6
TGF-beta	Transforming growth factor-beta
TGFBR2	Transforming growth factor-beta receptor type 2
TLR	Toll-like receptor
VAERS	Vaccine Adverse Event Reporting System
