# Supplementary figures and images for: Etiology Model of Kawasaki Disease and Multisystem Inflammatory Syndromes: Mast Cell Activation
Source: Curr Issues Mol Biol. 2026 May 14;48(5):508. doi: 10.3390/cimb48050508 (PMC13206014; doi:10.3390/cimb48050508)

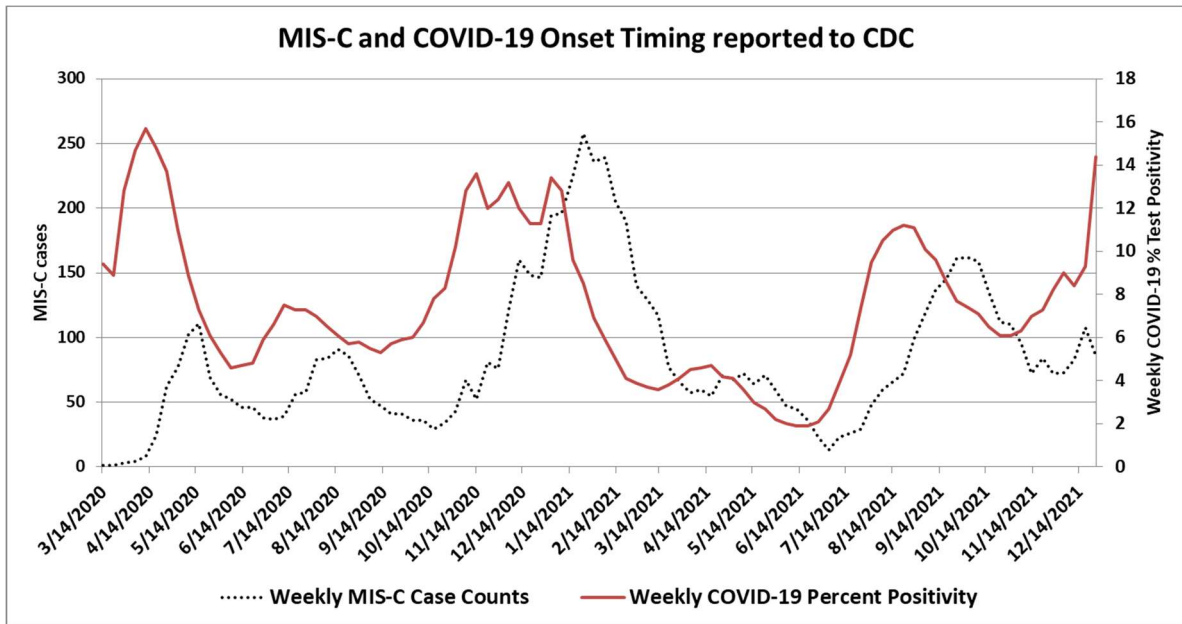

**Figure S1.** MIS-C reports track COVID-19 increases with delayed onset; data [166].

Supplement: Supplementary file 1 [file cimb-48-00508-s001.zip › Figure S1.pdf]
